# Clinical and Immunological Profile of Anti-factor H Antibody Associated Atypical Hemolytic Uremic Syndrome: A Nationwide Database

**DOI:** 10.3389/fimmu.2019.01282

**Published:** 2019-06-07

**Authors:** Mamta Puraswani, Priyanka Khandelwal, Himanshi Saini, Savita Saini, Bahadur Singh Gurjar, Aditi Sinha, Rajashri Pramod Shende, Tushar Kanti Maiti, Abhishek Kumar Singh, Uma Kanga, Uma Ali, Indira Agarwal, Kanav Anand, Narayan Prasad, Padmaraj Rajendran, Rajiv Sinha, Anil Vasudevan, Anita Saxena, Sanjay Agarwal, Pankaj Hari, Arvind Sahu, Satyajit Rath, Arvind Bagga

**Affiliations:** ^1^Division of Nephrology, Department of Pediatrics, All India Institute of Medical Sciences, New Delhi, India; ^2^Immuno Biology Laboratory II, National Institute of Immunology, New Delhi, India; ^3^National Centre for Cell Science, Pune, India; ^4^Regional Center for Biotechnology, Faridabad, India; ^5^Department of Transplant Immunology and Immunogenetics, All India Institute of Medical Sciences, New Delhi, India; ^6^Department of Pediatrics, BJ Wadia Hospital for Children, Mumbai, India; ^7^Department of Pediatrics, Christian Medical College, Vellore, India; ^8^Division of Pediatric Nephrology, Sir Ganga Ram Hospital, New Delhi, India; ^9^Department of Nephrology, Sanjay Gandhi Post Graduate Institute of Medical Sciences, Lucknow, India; ^10^Department of Pediatric Nephrology, Institute of Child Health and Hospital for Children, Madras Medical College, Chennai, India; ^11^Department of Pediatrics, Institute of Child Health, Kolkata, India; ^12^Department of Pediatric Nephrology, St. Johns Medical College and Hospital, Bengaluru, India; ^13^Department of Cardiology, All India Institute of Medical Sciences, New Delhi, India; ^14^Department of Nephrology, All India Institute of Medical Sciences, New Delhi, India; ^15^Translational Health Science and Technology Institute, Faridabad, India

**Keywords:** atypical hemolytic uremic syndrome, factor H, plasma exchange, renal reserve, thrombotic microangiopathy

## Abstract

**Background:** Atypical hemolytic uremic syndrome (aHUS), an important cause of acute kidney injury (AKI), is characterized by dysregulation of the alternative complement pathway. Autoantibodies to factor H (FH), a chief regulator of this pathway, account for a distinct subgroup. While high anti-FH titers predict relapse, they do not correlate well with disease activity and their functional characterization is required.

**Methods:** Of 781 patients <18-year-old of aHUS in the nationwide database from 2007 to 2018, 436 (55.8%) had anti-FH antibodies. Clinical features and outcome of patients managed in the last 6-year (*n* = 317) were compared to before (*n* = 119). In plasma samples of 44 patients, levels of serial circulating FH immune complexes (CIC), free FH, soluble terminal complement complex (sC5b-9), sheep red blood cell (SRBC) lysis and epitope specificity (*n* = 8) were examined. Functional renal reserve, ambulatory hypertension, left ventricular hypertrophy (LVH), and proteinuria were evaluated in a subset.

**Results:** Patients presented with markedly elevated anti-FH titers (10,633.2 ± 998.5 AU/ml). Management varied by center, comprising plasma exchange (PEX; 77.5%) and immunosuppression (73.9%). Patients managed in the last 6-year showed better renal survival at mean 28.5 ± 27.3 months (log rank *P* = 0.022). Mean anti-FH titers stayed 700–1,164 AU/ml during prolonged follow-up, correlating with CIC. Patients with relapse had lower free-FH during remission [Generalized estimating equations (GEE), *P* = 0.001]; anti-FH levels ≥1,330 AU/ml and free FH ≤440 mg/l predicted relapse (hazards ratio, HR 6.3; *P* = 0.018). Epitope specificity was similar during onset, remission and relapse. Antibody titer ≥8,000 AU/ml (HR 2.23; *P* = 0.024), time to PEX ≥14 days (HR 2.09; *P* = 0.071) and PEX for <14 days (HR 2.60; *P* = 0.017) predicted adverse renal outcomes. Combined PEX and immunosuppression improved long-term outcomes (HR 0.37; *P* = 0.026); maintenance therapy reduced risk of relapses (HR 0.11; *P* < 0.001). At 4.4±2.5 year, median renal reserve was 15.9%; severe ambulatory, masked and pre-hypertension were found in 38, 30, and 18%, respectively. Proteinuria and LVH occurred in 58 and 28% patients, respectively.

**Conclusion:** Prompt recognition and therapy with PEX and immunosuppression, is associated with satisfactory outcomes. Free-FH predicts early relapses in patients with high anti-FH titers. A significant proportion of impaired functional reserve, ambulatory hypertension, proteinuria and LVH highlight the need for vigilant long-term follow-up.

## Introduction

Hemolytic uremic syndrome (HUS) is an important cause of acute kidney injury (AKI) in children (1, 2). While the majority of patients follow gastrointestinal infection with Shiga toxin associated organisms, abnormalities in the complement and coagulation pathways are associated with atypical hemolytic uremic syndrome (aHUS) (1, 3). Although 5–25% patients in European cohorts show antibodies to factor H (FH) (4–6), this subset of illness is common in India accounting for ~50% cases (7). Recent data from the global aHUS registry, from centers in Europe, North America and Australia, confirm the presence of anti-FH antibodies in 24% children and 19% adults (8).

The pathogenesis of anti-FH associated aHUS and reasons for its high frequency in south Asia are unclear. While more than 80% patients show a homozygous deletion in the gene encoding FH related protein 1 (*CFHR1*), the deletion is present in 5–10% healthy people across the world. High levels of antibodies at disease onset or relapse are believed to induce functional deficiency of FH; their decline in response to plasmapheresis is associated with disease remission (7, 9, 10). The antibodies bind chiefly to the C-terminus of FH, inhibiting its cell surface regulatory functions (11, 12). A dose-response relationship is not established as many patients show high antibody levels even during remission, emphasizing the need to evaluate other markers of complement activation. Studies relating antibody titers to functional assays of FH inhibition, such as level of sheep red blood cell (SRBC) lysis, free FH, soluble terminal complement complex (sC5b-9) and epitope specificity of antibodies are limited (9, 13, 14).

We report the clinical features and outcomes of a large nationwide database of patients with anti-FH associated HUS. We also examined the functional implications of anti-FH antibodies and biomarkers that might enable prediction of a relapse.

## Methods

Since March 2007, 781 patients younger than 18-year-old with aHUS have been enrolled in a prospective multicenter nationwide database at the All India Institute of Medical Sciences (AIIMS). Of these, 436 patients with anti-FH antibody associated aHUS, diagnosed in presence of microangiopathic hemolytic anemia (hemoglobin < 10 g/dl, schistocytes ≥2%, lactate dehydrogenase >450 U/l), thrombocytopenia (platelets < 150,000/μl), AKI and anti-FH antibody titers >150 AU/ml (15) were included. Clinical features of patients enrolled until February 2013 have been reported earlier (7). Patients with septicemia, disseminated intravascular coagulation, and thrombotic microangiopathy secondary to medications, lupus, HIV infection, and following bone marrow transplantation were excluded. Institute ethics committee approval was obtained and informed written consent was taken prior to enrolment.

### Investigations

Anti-FH antibodies were screened in plasma samples by enzyme linked immunosorbent assay (ELISA) (7). Antibody titer, determined at serial dilutions, was expressed as arbitrary units (AU)/ml at 1:50 dilution; values >150 AU/ml were considered abnormal (7). Investigations included urinalysis, blood levels of complement C3, antinuclear antibody, and antineutrophil cytoplasmic antibody. Leptospirosis, dengue, malaria, and rickettsia might rarely mimic clinical and laboratory features of HUS; we therefore screened for these infections. Levels of anti-FH antibodies, creatinine and urinalysis were estimated every 3–6 months. Studies for functional effects of anti-FH antibodies, including circulating FH immune complexes (CIC), free FH, sC5b-9, and SRBC lysis, were performed in 44 consecutive patients managed at AIIMS. Epitope specificity of FH-antibodies was determined during onset, remission, and relapse, in a subset of eight patients.

#### Circulating FH Immune Complexes (CIC)

Briefly, 96-well plates (Nunc-Immuno Micro Well, Sigma-Aldrich, MO), coated with sheep anti-FH polyclonal antibody (AbD Serotec, Hercules, CA) diluted 1:10,000 in 0.5 M carbonate buffer (pH 9.6), were incubated overnight at 4°C (13). After washing, plates were blocked with 1% non-fat milk (Sigma-Aldrich) for 2-h at 37°C. Serial dilutions of plasma samples and controls were incubated for 1-hr, followed by incubation with goat anti-human IgG conjugated with horse radish peroxidase (HRP; Sigma-Aldrich) and titers reported at 1:100 dilution. For color development, H_2_O_2_ substrate and 3,3′,5,5′-tetramethylbenzidine were added; optical density was read at 450 nm. Titers of immune complexes, expressed as AU/ml, were calculated based on reference plasma (courtesy Marie Agnès Dragon-Durey). Screening of 100 healthy donors was used to establish the positive threshold of 110 AU/ml, corresponding to mean + 2 SD; intra- and inter-assay coefficient of variation was <10%.

#### Free Factor H

Plasma samples diluted to 1:100 were incubated with Protein G-coated beads (10:1; Sigma-Aldrich) for 1-h at room temperature, followed by centrifugation. The supernatant was used for CIC ELISA to confirm removal of all IgG-bound FH. ELISA plates, coated with sheep anti-FH polyclonal antibody (AbD Serotec) and blocked with 1% bovine serum albumin (BSA), were incubated for 1-h at room temperature with the supernatant diluted to 1:5000. The plate was washed and monoclonal anti-FH antibody (AbD Serotec) added, followed by washing and addition of anti-mouse IgG labeled with HRP with color development as described above. Purified FH (Calbiochem, Meudon, France) was used as the calibrator.

#### Sheep Red Blood Cell (SRBC) Hemolysis

Patient plasma was serially diluted to 1:1, 1:2, and 1:4 with buffer (20 mM Hepes, 7 mM MgCl_2_, 10 mM EGTA, 144 mM NaCl, 1% BSA; pH 7.4). Diluted plasma (10 μl) was incubated with 10 μl sheep erythrocytes (10^8^ cells/ml) at 37°C for 45 min [modified from (11)]. Following addition of 280 μl normal saline, the samples were centrifuged and absorbance read at 414 nm. Complete hemolysis with water was considered as 100%. Hemolysis was calculated as follows:

(1) $$\begin{array}{r} \text{SRBC lysis}=\frac{(\text{OD of patient sample}-\text{OD of blank})\;\times 100}{\text{OD of well labeled}100\%} \end{array}$$

Mean SRBC lysis in 20 healthy controls was 16.9±2.1%; the positive threshold was 21%.

#### Soluble Terminal Complement Complex (sC5b-9)

sC5b-9 was quantitated by ELISA using the MicroVue kit (Quidel Corp, San Diego, CA). Diluted plasma specimens were added to 96-well plate pre-coated with monoclonal antibody against C9 ring and incubated at room temperature for 1-hr. After addition of biotinylated antibody specific to sC5b-9, HRP-conjugated streptavidin and tetramethylbenzidine, optical density was read at 450 nm. sC5b-9 levels were calculated from a standard curve, limits of detection being 3.7–170 ng/ml.

#### Production of FH Fragments

Short consensus repeats (SCR) 1–4, 5–8, 9–12, 13–16, and 17–20 were PCR amplified with specific primers from the FH cDNA (OriGene, Rockville, MD) and cloned in pET28 and pET29 expression vectors (16). The clones were verified by DNA sequencing (ABI 3730 DNA analyzer; Applied Biosystems), products were transformed in *E. coli BL21* and expressed in inclusion bodies generated after induction with 1 mM isopropyl-1-thio-b-D-galactopyranoside and purified using Ni-NTA resin. The eluted fragments were purified by gel filtration using Superdex75 columns (GE Healthcare) and their concentration determined by Bradford assay (Bio-Rad).

#### Epitope Specificity

Plasma samples were incubated with FH fragments for 1-h and added to ELISA plates pre-coated with purified FH (Calbiochem) and blocked with 1% BSA. Bound antibodies were detected by HRP conjugated goat anti-human IgG with color development, as above.

### Therapy and Outcomes

The management of patients across centers was at the discretion of treating physicians, depending on their experience and available facilities. Renal replacement therapy was provided, when required. Specific management varied across centers and included plasma exchanges (PEX) and/or immunosuppressive therapies (9). Hematological remission was defined as platelet count >100,000/mm^3^, schistocytes <2% and LDH less than upper limit of normal on two consecutive days. Disease relapse was considered when there was a new episode of illness after the patient had achieved remission for ≥2 weeks. Outcomes at 3-months and last follow-up were assessed, in terms of estimated glomerular filtration rate (eGFR) (17), hypertension (18), dipstick proteinuria and relapses. Adverse outcome was defined as eGFR <30 mL/min/1.73 m^2^ on follow-up or patient death.

### Renal and Cardiovascular Outcomes

Long term outcomes were evaluated in 50 consecutive patients with eGFR more than 60 ml/min/1.73 m^2^ and two or more years' follow-up. Blood pressure was recorded and classified using standard guidelines (18). First morning urine protein-to-creatinine ratio >0.15 mg/mg was considered abnormal (19). On echocardiography, left ventricular mass index (LVMI) was defined as ratio of left ventricular mass to height^2.7^; LVH was LVMI >95th centile for age and sex (20). Dyslipidemia was defined as fasting total cholesterol >170 mg/dl, LDL cholesterol >110 mg/dl, non-LDL cholesterol >120 mg/dl, triglycerides >75 mg/dl or HDL cholesterol <40 mg/dl (21).

#### Ambulatory Blood Pressure Monitoring (ABPM)

Ambulatory blood pressure was recorded by oscillometry (90207, Spacelabs Medical, Redmond, WA). Data was reported as standard deviation scores (SDS) based on height and sex specific normative data (22). Patients were classified as having ambulatory hypertension if they showed clinic hypertension (blood pressure ≥95th percentile), mean ambulatory systolic or diastolic blood pressure exceeding 95th percentile for sex and height, and systolic or diastolic load >25% (severe if load >50%) (22). Masked hypertension was defined as ambulatory hypertension with normal clinic blood pressure (22).

#### Functional Renal Reserve

Functional renal reserve was evaluated following administration of oral trimethoprim (10 mg/kg/day) for 5 days (23), discontinuation of ACE-inhibitors, and a vegetarian diet for 48-h (24). Patients voided completely at 7:00 am on the day of test; residual urine, estimated by ultrasonography, was required to be below 10 ml. They were instructed to drink 5 ml/kg water every 30 min throughout the study period. An accurately timed urine collection of 2-h duration was obtained and residual urine rechecked; serum creatinine was estimated at its midpoint. A protein meal (1 g/kg; RiteBite Max bar containing 20 g casein and whey protein) was ingested. Following complete voiding, 40 min later, similar 2-h urine collection and measurement of serum creatinine were done. Renal clearance of creatinine (CrCl) and functional renal reserve were calculated:

(2) $$\begin{array}{r} \text{CrCl}=\frac{\text{UCr}\times \text{V}}{\text{sCr}\times \text{t}}\times \frac{1.73}{\text{BSA}} \end{array}$$

UCr urine creatinine, V urine volume over 2-h, *t* duration of collection (minutes), BSA body surface area, m^2^

(3) $$\begin{array}{r} \text{Functional renal reserve}=\frac{\text{(CrCl after protein load-Baseline CrCl)}}{\text{Baseline CrCl}}\times 100 \end{array}$$

### Statistical Analysis

Six-year cohorts of March 2007 to December 2012 and January 2013 to August 2018 were compared in terms of clinical features and outcomes. Data is presented as proportions, and median (interquartile range, IQR) or mean ± SD, based on distribution and analyzed using Stata version 14.0 (Stata Corp, College Station, TX). Anti-FH antibodies were expressed as mean ± SEM. Tests for significance included *t*-test, Wilcoxon signed rank and rank sum tests, and chi-square test; correlation was measured by Spearman coefficient. Anti-FH and free FH levels were log transformed to satisfy normality. Repeated measures analyses by generalized estimating equations (GEE) approach was used to compare serial anti-FH and free FH concentrations in sustained remission or subsequent relapse; receiver operator characteristic (ROC) curves were used for threshold of relapse. Determinants of adverse outcome and relapse were estimated as odds and hazards ratios, by univariate and multivariable analyses. Functional renal reserve was normalized by Box-Cox transformation. Linear regression analyses were used to evaluate predictors of renal reserve, LVMI and proteinuria; two tailed *P* < 0.050 was considered significant.

## Results

From March 2007 to August 2018, 436 (55.8%) of 781 patients from 30 centers in the nationwide database were diagnosed as having anti-FH associated aHUS (Supplementary Figure 1). Proportion of patients with anti-FH antibodies younger than 4-year, between 4–11 and 11–18 year at presentation were 20.8, 73.8, and 52.0%, respectively; five patients presented in infancy (Supplementary Figure 2). Patients between 4 and 11 year had higher antibody titers (11,127 ± 1,170 AU/ml vs. 8,870 ± 1,890 AU/ml; *P* = 0.025). There was seasonal variation, with peak between December and April (Supplementary Figure 3). Prodromal illness included fever (54.6%), upper respiratory tract infection (10.3%), and diarrhea (6.7%). Eight of 282 patients (2.8%) showed antinuclear antibodies, 3 of 219 (1.4%) had antineutrophil cytoplasmic antibodies. Three of 197 (1.5%) showed antibodies to leptospira; vivax or falciparum malaria was present in fifteen.

Five adult patients (aged 22–48 years) also showed anti-FH antibodies (854–60,032 AU/ml) and presented with similar clinical features, chiefly following a febrile illness. One patient each with systemic lupus erythematosus and following bone marrow transplant also showed elevated levels (852 and 4,264 AU/ml, respectively). These seven-patients have not been included to maintain homogeneity within a pediatric aHUS population.

Table 1 shows that patients presented earlier during the illness in the last 6-year compared to before 2013; stage 2 hypertension and seizures were also fewer in the latter cohort. Sixteen (3.6%) patients did not have thrombocytopenia, including six with mildly deranged renal function. Neurological features (31.3%) comprised seizures and/or hypertensive encephalopathy (12.4%). Imaging showed posterior reversible encephalopathy (*n* = 15), infarcts (*n* = 7) and intracranial hemorrhage (*n* = 2). Other features included cardiogenic shock (*n* = 16), pulmonary edema and hemorrhage (*n* = 8), and pancreatitis, mesenteric ischemia and peripheral gangrene (*n* = 3, each).

**Table 1:** Clinical and biochemical features in patients with anti-FH associated hemolytic uremic syndrome in two 6-year cohorts.

**Variable**	**2007-12 (*n* = 119)**	**2013-18 (*n* = 317)**	**Whole cohort (*n* = 436)**	***P***
Boys	91 (76.5)	211 (66.6)	302 (69.3)	0.048
Age, years	7.9 ± 3.6	7.6 ± 3.2	7.7 ± 3.3	0.26
Time to presentation, days^a^	18.0 ± 18.2	12.4 ± 12.1	13.8 ± 14.1	0.001
Duration of oligoanuria, days	11.7 ± 11.2	6.7 ± 9.9	8.0 ± 4.5	< 0.001
Anuria	52 (43.7)	79 (24.9)	131 (30.0)	< 0.001
Prodromal illness				
Febrile illness	63 (52.9)	175 (55.2)	238 (54.6)	0.74
Diarrhea, dysentery	10 (8.4)	19 (6.0)	29 (6.7)	0.24
Upper respiratory tract infection	6 (5.0)	39 (12.3)	45 (10.3)	0.032
Jaundice, elevated transaminases	24 (20.2)	138 (43.5)	162 (37.2)	< 0.001
Seizures	46 (38.7)	71 (22.4)	117 (26.8)	< 0.001
Stage 2 hypertension	84 (70.6)	154 (48.6)	238 (54.6)	< 0.001
Hemoglobin, g/dl	5.5 ± 1.3	5.2 ± 1.3	5.3 ± 1.3	0.67
Platelet count, × 10^3^/mm^3^	63.9 ± 39.3	58.5 ± 39.1	59.9 ± 39.1	0.57
Reticulocyte count, %	11.0 ± 9.5	8.7 ± 6.6	9.2 ± 7.3	0.10
Nephrotic range proteinuria	36 (30.3)	211 (66.6)	247 (56.7)	< 0.001
Blood creatinine, mg/dl	5.84 ± 2.67	5.46 ± 3.08	5.56 ± 2.98	0.28
Lactate dehydrogenase, IU/L	3042.0 ± 2701.4	3582.5 ± 2873.1	3,447 ± 2837.4	0.005
Complement C3, mg/dl	70.0 ± 28.6	71.2 ± 27.9	70.9 ± 28.1	0.84
Anti-FH antibody, AU/ml^*^	7330.5 ± 2017.4	11847.4 ± 1,140.1	10,633.2 ± 998.5	0.005

*Normal range: hemoglobin 11.0–15.8 g/dl, platelet count 190–590 × 10^3^/mm^3^, reticulocyte count 3.6–11%, blood creatinine: 0.17–1.01 mg/dl, lactate dehydrogenase 195–314 IU/L, C3 90–130 mg/dl*.

a *Days between disease onset and evaluation*.

Data shown as N (%), mean ± standard deviation or

* *mean ± standard error of mean; AU arbitrary units; FH factor H*.

### Anti-FH Titers and Their Functional Characterization

**Figure 1:**
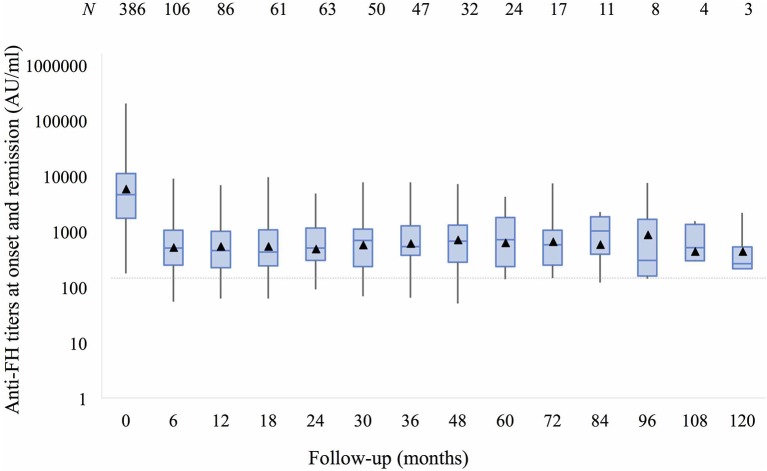
Anti-factor H antibodies (shown as mean titers, ▴) at onset and 6–12 monthly follow-up over 10 years remain detectable (>150 AU/ml) despite remission. Boxes depict median and interquartile range; whiskers show range of antibody titers at each follow-up visit.

**Figure 2:**
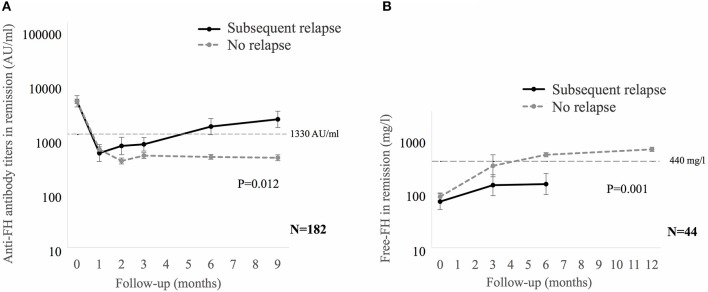
**(A)** Mean anti-factor H (FH) antibody titers, and **(B)** Free FH during remission in patients with or without subsequent relapse. Relapses were seen in 12 of 182 **(A)** and 10 of 44 **(B)** patients. Generalized estimating equations approach was used on log transformed data. In patients with relapse, the last anti-FH titer and free FH was estimated at median of 1 (0.7–2) months and 5 (2–6.9) months before relapse, respectively. Anti-FH antibody titers ≥1,330 AU/ml or free FH ≤ 440 mg/l predict later relapse.

Anti-FH titers at onset negatively correlated with serum C3 (*P* < 0.001), platelets (*P* = 0.013), and hemoglobin level (*P* = 0.057), and positively with LDH (*P* = 0.010; Supplementary Figure 4). Patients requiring dialysis had higher mean antibody titers than those not dialyzed (11,287 ± 1,173 vs. 8,198 ± 2,017 AU/ml; *P* = 0.015). Figure 1 shows that anti-FH titers remained detectable during remission, with mean titers varying between 700 and 1,164 AU/ml on follow-up. Mean anti-FH titers during remission in patients with or without subsequent relapse are shown in Figure 2A. Using GEE approach, we found that patients with relapses had significantly higher antibody titers 1 month preceding a relapse compared to those in sustained remission (ß = 0.18; *P* = 0.023). ROC curves showed that titers ≥1,330 AU/ml at 6-months predicted the occurrence of a relapse (sensitivity 75%, specificity 81.4%; area under curve 0.86). However, 27 (15.8%) patients with sustained remission had antibody levels above this cut-off.

Table 2 shows serial levels of CIC, free FH, C3, sC5b-9 and SRBC lysis from onset to 12-months in 44 patients. While median CIC declined from 20,000 AU/ml at onset to 806 AU/ml at remission (*P* < 0.001), it remained detectable and correlated with antibody titers at 6- and 12-months (*r* = 0.44 and 0.40; *P* = 0.007 and 0.067, respectively). SRBC lysis reduced from 72.8% at onset to 16.9% during remission, and levels of free FH increased from 64 to 553 mg/l. SRBC lysis and free FH correlated with CIC (*r* = 0.68 and *r* = −0.63, *P* < 0.001) and anti-FH titers (*r* = 0.60 and *r* = −0.55, *P* < 0.001) at all times points. While blood levels of sC5b-9 declined significantly during remission compared to onset (Table 2), they were high compared to controls (*P* < 0.001).

**Table 2:** Functional characterization of anti-factor H antibodies at onset and during remission during first year of follow-up.

	**Onset (*N* = 44)**	**Remission**		
		**3-months (*N* = 42)**	**6-months (*N* = 37)**	**12-months (*N* = 23)**
Anti- FH antibody, AU/ml	5,000 (2,123–163,829)	409 (254–861)	277 (154–893.6)	408 (262–691.8)
Circulating FH immune complexes, AU/ml	20,000 (7,168–44,480)	806 (289–1,328)	710 (244–1,681)	1,004.5 (397–1,663)
Sheep red blood cell hemolysis, %	72.8 (57.2–88.7)	16.9 (13.5–22.6)	14.6 (13–19.1)	13 (5.4–20)
Free FH, mg/l	64 (34–106)	–	553 (376–630)	779 (571–1,071)
Soluble terminal complement complex (sC5b-9), ng/ml	1,510 (832–2,220)	–	351 (260–720)	355.5 (232.5–642.5)
Complement C3, mg/dl	60.7 (48.7–82.6)	–	108.9 (95.5–129.9)	121.9 (94.4–130.2)

*P < 0.001 for all comparisons from onset*.

*Values are median (interquartile range); AU, arbitrary units; FH, factor H*.

*Normal ranges: Anti-FH antibody <150 AU/ml; circulating FH-immune complexes <110 AU/ml; sheep red blood cell hemolysis 16.9 ± 2.1%; median free FH 720 (459–810) mg/l; median sC5b-9 195.3 (151.1–292.5) ng/ml; C3 90–130 mg/dl*.

Since anti-FH titers ≥1,330 AU/ml were a predictor of relapse, we examined levels of free FH, sC5b-9 and CIC in patients with sustained remission but having anti-FH titers persistently above (*n* = 11) or below (*n* = 33) this cut-off. Median sC5b-9 and CIC were 709 ng/ml and 2,196 AU/ml in the former vs. 329.9 ng/ml and 594 AU/ml (*P* = 0.067 and *P* = 0.060, respectively); levels of free FH were also similar (*P* = 0.66).

We also examined serial free FH levels during remission in 44 patients, including 10 who later relapsed (Figure 2B). Free FH levels were significantly lower at 6-months in patients who relapsed compared to those in sustained remission (ß = 0.29; *P* = 0.001). ROC curves showed that free FH ≤ 440 mg/l at 6-months predicted occurrence of relapse (sensitivity 70%, specificity 100%; area under curve 0.91). Among patients with anti-FH levels ≥1,330 AU/ml, free FH ≤ 440 mg/l at 6-months discriminated between patients with a relapse and those with sustained remission, with sensitivity of 75%, positive predictive value of 79% and negative predictive value of 91%; area under curve = 0.91; hazards 6.3, 95% CI 1.7–23.8 (*P* = 0.018).

Epitope specificity (*n* = 8 patients) showed similar pattern of antibody binding during onset, remission and relapse. Antibodies demonstrated strongest binding to SCR 17–20 (*n* = 8), moderate binding to SCR 9–12 and SCR 13–16 (*n* = 7, each), and also to SCR 1–4 (*n* = 3), and SCR 5–8 (*n* = 4; Supplementary Table 1).

### Therapy

PEX was initiated earlier (median 11 vs. 17 days from onset of illness) and dialysis requirement was briefer (median 13 vs. 26 days) in the last 6-year (Table 3). PEX were done for at least 14 days in 72.7%; 42 patients received plasma infusions only. Based on center preference, initial immunosuppression comprised prednisolone with IV cyclophosphamide (*n* = 171) or rituximab (*n* = 43). Maintenance immunosuppression included prednisone alone (16), and with mycophenolate mofetil (133) or azathioprine (40).

**Table 3:** Therapy and outcomes in patients with anti-factor H associated hemolytic uremic syndrome.

**Variable**	**2007-12 (*n* = 119)**	**2013-18 (*n* = 317)**	**Whole cohort (*n* = 436)**	***P***
Dialysis requirement (%)	101 (84.9)	236 (74.4)	337 (77.3)	0.021
Duration of dialysis, days	26 (10–57)	13 (5.8–30.3)	15 (6–36)	< 0.001
Plasma exchange (PEX, %)	91 (76.5)	247 (77.9)	338 (77.5)	0.75
Days to PEX	17 (7–32)	11 (6–22)	12 (6–24)	0.011
Induction immunosuppression (%)	79 (66.4)	243 (76.7)	322 (73.9)	0.029
Maintenance immunosuppression (%)	50 (42)	139 (43.8)	189 (43.3)	0.73
Days to immunosuppression	34 (20–53)	21 (11–31.8)	27 (12–35)	< 0.001
Onset to hematological remission, days	38 (22.5–54.3)	24 (16–35)	27 (17–41)	< 0.001
**Outcome at 3-months**	***N*** **=** **105**	***N*** **=** **251**	***N*** **=** **356**	
Stage 2 HTN or proteinuria ≥2+	48 (45.7)	104 (41.4)	152 (42.7)	0.46
CKD stages 2–3	18 (17.1)	46 (18.3)	64 (18.0)	0.79
Adverse outcome CKD stage 4–5; death	35 (33.3)	46 (18.3)	81 (22.8)	0.002
**Outcome at last follow-up**	***N*** **=** **105**	***N*** **=** **251**	***N*** **=** **356**	
Stage 2 HTN or proteinuria ≥2+	31 (26.1)	64 (20.2)	95 (26.7)	0.43
CKD stages 2–3	12 (10.1)	27 (8.5)	39 (11.0)	0.86
Adverse outcome CKD stage 4–5; death	38 (36.2)	53 (21.1)	91 (25.6)	0.003
Relapse	26 (24.7)	35 (13.9)	61 (17.1)	0.003

*Data shown as median (interquartile range) or N (%); CKD, chronic kidney disease; HTN, hypertension; PEX, plasma exchange*.

### Outcome

**Figure 3:**
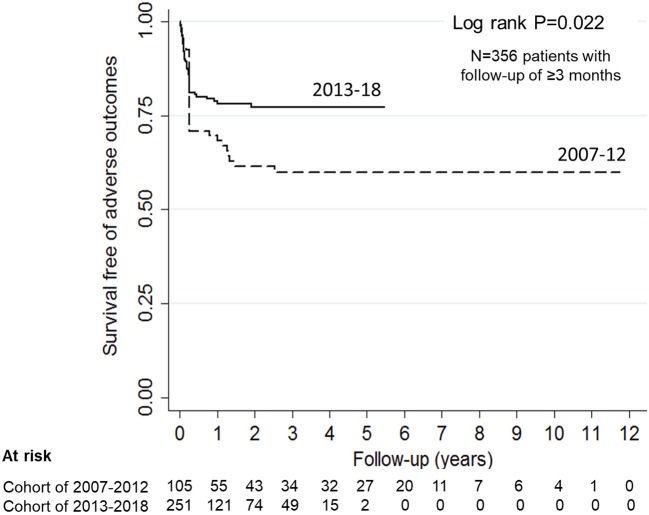
Probability of renal survival in patients with anti-factor H antibody associated HUS. Patients diagnosed and managed from 2007 to 12 showed 70.8, 59.8, and 59.8% renal survival at 1-, 5-years and at last follow up (interrupted line). Corresponding renal survival in patients managed during 2013-18 (continuous line) was 78.7, 77.1, and 77.1% (log rank *P* = 0.022).

Information on outcome at 3-months or longer was available for 356 (81.7%) patients. During the last 6-year, patients achieved earlier hematological remission and showed better outcomes at 3-months (Table 3) and last follow-up (Figure 3). In the subset of 196 patients, managed with PEX and immunosuppression, adverse outcomes occurred in 12.2% at 3-months (Supplementary Table 2); survival free of adverse outcomes was 86, 86, and 78% at 1-year, 5-year and last-follow up at mean 28.5 ± 27.3 months, respectively. Sixty-one (17.1%) patients relapsed at mean 11.0 ± 12.9 months from onset; relapse free survival was 86.4, 73.5, and 68.3% at 1-year, 5-year and last follow up, respectively. Relapses were early, with 46% occurring within the first 6-months and 95% within 2-years.

### Determinants of Outcome

Mean antibody titer was 22,801 ± 6,712 AU/ml in 19 patients who died in the first month of illness compared to 9,851 ± 1,058 AU/ml in those who survived (*P* = 0.002). On multivariable analysis, antibody titer at onset ≥8,000 AU/ml (OR 6.1, 95% CI 0.88–43.0; *P* = 0.066) predicted mortality within 30 days of onset, while daily PEX for 7 days (OR 0.14, 95% CI 0.02–0.95; *P* = 0.044) and concomitant immunosuppression (OR 0.07, 95% CI 0.01–0.71; *P* = 0.024) were protective (data not shown). Independent predictors of adverse outcome were antibody titer ≥8,000 AU/ml (HR 2.23, 95% CI 1.11–4.48; *P* = 0.024), longer time (≥14 days) between onset of illness and initiation of PEX (HR 2.09, 95% CI 0.93–4.69; *P* = 0.071), and short duration PEX < 14 days (HR 2.60, 95% CI 1.19–5.67; *P* = 0.017). Therapy with PEX and induction therapy (HR 0.37; 95% CI 0.16–0.89; *P* = 0.026) and maintenance immunosuppression (HR 0.02; 95% CI 0.001–0.413; *P* = 0.011) protected against adverse outcomes (Supplementary Table 3). Maintenance immunosuppression was associated with relapse free survival (HR 0.11; 95% CI 0.05–0.27; *P* < 0.001).

### Renal, Cardiovascular Outcomes

Fifty consecutive patients with eGFR 100.2 ± 21.1 (range 67–156) ml/min/1.73 m^2^ were screened for renal and cardiovascular outcomes at mean 4.4 ± 2.5 (range 2–15) year from onset. Seventeen patients had CKD stage 2 and proteinuria was present in 29. Despite 37 patients receiving antihypertensive agents, 24 had clinic hypertension. ABPM showed normal blood pressure (14%), severe ambulatory hypertension (38%), masked hypertension (30%), pre-hypertension (18%), and abnormal nocturnal dipping (76%). Mean systolic and diastolic blood pressures were at 77th and 87th percentile with mean load 43 ± 26 and 40 ± 26%, respectively. LVH was present in 28% and 5 patients had dyslipidemia.

Median renal reserve was 15.9 (6.3, 28.5)% in 41 patients (Supplementary Figure 5). Proportion of patients with renal reserve below 20, 10, and 5% were 49, 27, and 15%, respectively. Renal reserve was 10.3 (−5.3, 31.5)% in patients with stage 2 CKD compared to 27.9 (16.9, 40.4)% with eGFR >90 ml/min/1.73 m^2^ (*P* = 0.010). Factors independently and inversely associated with renal reserve were mean systolic ambulatory pressure (ß = −0.49, 95% CI −0.85,−0.12; *P* = 0.010), number of relapses (ß = −0.65, 95% CI −1.23, −0.07; *P* = 0.030), urine protein-to-creatinine ratio >1 (ß = −0.9, 95% CI −0.1, −1.71; *P* = 0.031) and increased LVMI (ß = −0.04, 95% CI −0.08, −0.001; *P* = 0.044). Proteinuria was associated with ambulatory systolic (ß = 0.19, 95% CI 0.07, 0.32; *P* = 0.003) and diastolic hypertension (ß = 0.16, 95% CI 0.03, 0.3; *P* = 0.021).

## Discussion

We report the clinical features and outcomes of a large, prospective multicenter cohort of 436 children with anti-FH associated aHUS managed across the country over a period of 12-year. Although the management was heterogeneous and based on center practices and physician preference, patients managed in the recent years show overall better renal survival, with decline in proportion of adverse outcomes from 36.2 to 21.1%. Prompt PEX, performed for at least 2-weeks, combined with immunosuppressive medications resulted in renal survival of 86% at 5-year. Serial examination showed that anti-FH titers and circulating FH immune complexes persisted well above the normal range during remission. Among patients with high anti-FH level exceeding 1,330 AU/ml, level of free FH below 440 mg/l predicted a 6.3-fold risk of later relapse. On long-term follow-up, patients show significant sequelae in terms of severe and masked ambulatory hypertension, left ventricular hypertrophy and proteinuria (summarized in Table 4).

**Table 4:** Summary of key findings in patients with anti-factor H (FH) associated hemolytic uremic syndrome (HUS).

**Objective, *N***	**Result**	**Conclusion**
Demographic features, *N* = 436	Of 781 patients < 18-years-old, 55.8% had anti-FH antibodies. Cases peak between December and April; prodrome: fever (54.6%), upper respiratory tract infection (10.3%), diarrhea (6.7%)	Seasonal predilection and prodromal symptoms indicate possible infectious trigger
Cohorts: 2007-12 (*N* = 119); 2013-18 (*N* = 317)	Earlier diagnosis and initiation of therapy in cohort of 2013-18; better outcomes at 3-months (33.3 vs. 18.3%) and at last follow-up (*P* = 0.022) in recent years	Prompt recognition and appropriate management improves outcomes
Anti-FH antibody titer and impact on course	Anti-FH titers at onset negatively correlate with serum C3, platelets and hemoglobin level; positive correlation with LDH levels and need for dialysis. Mean anti-FH titers 700-1164 AU/ml over 10-year follow up Anti-FH titers ≥1,330 AU/ml at 6-months predicts relapse (sensitivity 75%, specificity 81.4%; AUC 0.86); 15.8% patients in remission show antibody levels >1,330 AU/ml	Anti-FH antibody titer correlates with disease severity at onset. Titers high in remission; need biomarkers to predict relapse. Patients with anti-FH titers >1,330 AU/ml at risk of relapse—require careful clinical monitoring.
Functional characterization of antibodies, *N* = 44	Circulating FH immune complexes (CIC) decline but correlate with anti-FH titers during remission. During remission, median soluble terminal complement complex (sC5b-9) levels were 329.9 ng/ml and 594 ng/ml in patients with high or low titers, respectively.	CIC and sC5b-9 elevated even during remission; unsatisfactory biomarkers of disease
	Free FH ≤ 440 mg/l at 6-months predicts relapse (sensitivity 70%, specificity 100%; AUC 0.91). Presence of free FH ≤ 440 mg/l *and* antibody ≥1,330 AU/ml associated with 6.3-fold risk of relapse	Low levels of free FH predict relapse; requires examination in a larger cohort
FH epitope specificity, *N* = 8	Similar binding during onset, relapse, remission. Strong binding to SCR 17–20; also to others	Binding at multiple epitopes on FH
Outcome, *N* = 356	Independent predictors of adverse outcome: Anti-FH ≥8,000 AU/ml, long time to begin PEX (>14 days from onset) and short duration PEX (< 14 days); combined PEX and immunosuppression were protective. Maintenance immunosuppression reduces risk of relapses.	Antibody titers at onset predict early mortality and outcomes. Adequate PEX with immunosuppression improve outcomes.
Outcomes at 4.4 ± 2.5 year from onset, *N* = 50	eGFR 100.2 ± 21.1 ml/min/1.73 m^2^; proteinuria (58%), severe ambulatory hypertension (38%), masked (30%), prehypertension (18%), left ventricular hypertrophy (28%), and dyslipidemia (10%).	More than one-third patients show renal and cardiovascular sequelae
Renal reserve, *N* = 41	Median renal reserve 15.9%. Inverse association with mean systolic pressure, number of relapses, urine protein-to-creatinine ratio, and increased left ventricular mass index.	Suggest long term assessment for proteinuria, ambulatory hypertension, cardiovascular outcomes

*AUC, area under the curve; eGFR, estimated glomerular filtration rate; FH, factor H; LDH, lactate dehydrogenase; PEX, plasma exchange; SCR, short consensus repeats; sC5b-9, soluble terminal complement complex*.

While 24% children and 19% adults in the global aHUS Registry had anti-FH antibodies (8), these autoantibodies were present in 55.8% patient in the current cohort, confirming the increased frequency of this condition in India (7). In conformity with previous reports, predominantly children between 4 and 11-years were affected (4, 8, 25). The reason for high prevalence of anti-FH antibody associated HUS in Indian children is not clear. The population frequency of homozygous *CFHR1* deletion is similar in India (9.5%) compared to elsewhere (2–10%) (7, 9). The high prevalence of the illness in school going children, predilection for the cold weather, and associated prodromal symptoms indicate a possible infectious trigger. While a gastrointestinal prodrome is reported by others (1, 25), the chief preceding illness in the present patients was low-grade fever (55%) or a respiratory tract infection. A previous study from this center, using multiplex polymerase chain reaction on stool specimens, showed multiple gastrointestinal pathogens in 35 patients predominantly in patients with anti-FH antibodies (26).

Anti-FH titers were related to disease severity as evidenced by relationship with platelet count, hemoglobin, blood levels of LDH, dialysis requirement, mortality, and renal outcome. As shown previously, we found that CIC and SRBC lysis were markedly elevated during active disease compared to remission (12, 13, 27–29); the decline in CIC during remission was more than that of free antibodies, perhaps due to change in avidity of antibodies for free FH (13). While our finding of persistently high antibodies during remission has been reported (4, 25), the present report emphasizes that levels of CIC continued to be high during follow-up. Other authors suggest that CIC correlate better with disease activity, than do antibody titers (13).

The present analysis confirms our previous findings on the association of high antibody titers (≥1,330 AU/ml at 6-months) with subsequent relapse (30). However, 15.8% patients with quiescent disease had persistently high titers, suggesting that elevated levels are not always pathogenic. While free FH has been measured in few patients (31, 32), we determined serial levels in a larger cohort. Among patients with high anti-FH titer, reduced free FH concentration ( ≤ 440 mg/l) predicted a 6.3-fold higher risk of subsequent relapse with a negative predictive value of 91%. These findings suggest that formation of CIC reduce availability of free FH, impairing cell surface protection. This was shown previously *in vitro* as dose-dependent reduction in SRBC lysis on addition of FH to sera of anti-FH positive patients (11). Our findings suggest that estimation of free FH is therefore promising for indicating the risk of relapses.

Previous studies on levels of sC5b-9, which assesses activity of the soluble terminal complement pathway, show variable levels during active disease and remission (29, 33–36). While all the present patients had elevated sC5b-9 at onset that declined during remission, the levels were higher than normal, as shown previously in some but not all studies (29, 36). Serial estimation of blood levels of sC5b-9 did not therefore predict relapses, limiting its utility as a biomarker of disease activity. In contrast, free FH levels were normal in patients with sustained remission but having high anti-FH antibody titers, suggesting its potential role as a biomarker.

While free FH is presently a research tool and not available widely, close monitoring of anti-FH antibody titers is required with more careful assessment if elevated >1,300 AU/ml during the first 12–24 months (37). Relapses usually follow minor infectious illnesses during which close clinical and biochemical monitoring is required.

Using FH fragments that were generated in *E. coli* and purified by gel filtration, Gurjar et al. used inhibition ELISA to determine epitope specificity in 21 patients of this cohort with anti-FH associated HUS (16). Antibodies showed strong binding to SCR 17–20; binding with lower affinity was present to SCR 5–8 (16). We extended this work to examine whether there was altered epitope specificity to FH at onset of the illness, remission and during relapse in eight more patients. Similar to the previous work, we found binding to SCR 17–20 in all patients at onset; binding to SCR 9–12 and SCR 13–16 was also present in most patients. There were no significant changes in epitope binding between onset, remission and relapse, as shown previously (14). The small number of patients studied limits conclusions regarding epitope specificity. Table 5 summarizes findings from various reports on epitope specificity of anti-FH antibodies, emphasizing predominant binding to the C-terminal, and also other domains on FH (5, 11, 13, 14, 16, 25, 38–40).

**Table 5:** Epitope specificity of anti-factor H (FH) antibodies to short consensus repeats (SCR) of FH.

**References**	**SCR 1–4**	**SCR 1–7**	**SCR 7**	**SCR 5–8**	**SCR 8–14**	**SCR 9–12**	**SCR 11–14**	**SCR 13–16**	**SCR 15–20**	**SCR 17–20**	**SCR 19–20**
Blanc et al. (13)	13/14	17/18			5/18				18/18		8/17
Bhattacharjee et al. (38)									10/10		10/10
Moore et al. (39)	1/12										7/12
Jozsi et al. (11)		0/5			1/5				5/5		5/5
Jozsi et al. (5)		0/16			4/16				16/16		16/16
Nozal et al. (14)	1/14	1/14			2/17				12/14		
Guo et al. (40)	4/36		6/36				4/36				12/36
Gurjar et al. (16)				21/21						21/21	
Brocklebank et al. (25)		5/17			1/17^#^				1/17^*^		15/17
Present study	3/8			4/8		7/8		7/8		8/8	
Total (%)	$\underset{︸}{35.4\%(81/229)}$				$\underset{︸}{24.8\%(31/125)}$				$\underset{︸}{73.9\%(164/222)}$		
	N-terminal of FH				Mid-portion of FH				C-terminal of FH		

# Short consensus repeats (SCR) 8–15;

* *SCR 16–18*.

Education and dissemination of management protocols through scientific meetings and efforts for consensus guidelines (37) has resulted in prompt recognition, early referral, and protocol based management, improving patient outcomes. While PEX and immunosuppression are considered primary therapies for patients with anti-FH associated HUS, the duration of therapy is empirical (41). The present report suggests that prompt and adequate duration of PEX was associated with decreased mortality and improved renal outcomes. Since most relapses occurred in the initial 2 years, immunosuppression is recommended for this period. We did not find increased rates of infections with use of immunosuppressive agents, as was a concern in a previous study (25). Our findings also suggest that strategies like PEX with corticosteroids alone, or immunosuppression with/without plasma infusions had limited benefit on long-term outcome.

An audit on safety of PEX from centers in Europe and North America showed procedure related complications and hypersensitivity to plasma in one-third patients, limiting the safety of this procedure in children (42). A similar audit of 2024 PEX sessions in 109 patients in New Delhi showed chiefly self-limiting adverse events (9.1%), including chills, vomiting, abdominal pain, and urticaria; hypotension (1.6%), hypocalcemia, tachycardia, seizures (0.2%, each), and hemorrhage (0.1%) were rare and catheter-related adverse events comprised only bloodstream infection (1.45/1,000 catheter-days). Hematological remission was achieved in 93.4% of patients within a fortnight of initiating PEX, with 80% and 90% patients discontinuing dialysis by 1 and 3 months, respectively (43). PEX was therefore overall safe and effective with satisfactory short-term outcomes.

Inhibition of the complement pathway with eculizumab is the standard of care for aHUS in developed countries (15). Most patients with anti-FH associated illness are treated similarly (25), although international pediatric and KDIGO guidelines suggest the use of PEX and immunosuppressive therapy for this disorder (15, 44). However, eculizumab does not impact generation of antibodies and additional immunosuppression might still be required. On the other hand, present findings show that despite PEX and immunosuppressive therapy almost one-quarter of all patients with anti-FH associated HUS had an adverse outcome. Some of these patients might have benefited from prompt use of eculizumab, especially if hematological remission was delayed beyond 7–10 days. There is need for a prospective study examining the efficacy and safety of eculizumab in this specific condition.

The rates of persistent proteinuria (15–30%) and ambulatory hypertension (10–46%) following Shiga toxin HUS are similar to the present cohort (45–48). Masked hypertension and abnormal dipping of blood pressure are proposed to be associated with adverse cardiovascular outcomes and microalbuminuria (49, 50). The findings of abnormal dipping in three-quarter of all patients, and masked hypertension, LVM in one-third are therefore important. We also found reduced renal functional reserve in one-third patients, similar to 24–65% in patients with Shiga toxin HUS (51–54). Conforming to previous reports, functional reserve was inversely associated with proteinuria and ambulatory hypertension (53, 55–57). Since we assessed functional reserve in patients with eGFR >60 ml/min/1.73 m^2^, its overall magnitude is perhaps higher. The present findings emphasize that patients recovering from anti-FH associated HUS require long-term assessment for cardiovascular and renal outcomes.

## Data Availability

The datasets generated for this study are available on request to the corresponding author.

## Ethics Statement

Institute ethics committee approval was obtained from All India Institute of Medical Sciences, New Delhi and informed written consent was taken prior to enrolment.

## Author Contributions

MP: performed experiments, manuscript preparation. PK: patient care, data collection and analysis, manuscript preparation. HS, BG, RS, TM, AKS, and SS: laboratory work for the study. AnS, SA, AdS, and PH: patient care, critical review of manuscript. UA, IA, KA, NP, PR, RS, and AV: patient care. ArS, SR, UK, and AB: supervision of experiments. AB: study design, patient care, manuscript preparation and also is the guarantor for this paper. All authors approved the manuscript before it was submitted.

## Contributing Centers

Indira Agarwal, Christian Medical College, Vellore; Vinay Aggarwal, BL Kapoor Hospital, New Delhi; Uma Ali, Bai Jerbai Wadia Hospital for Children, Mumbai; Kanav Anand, Sir Ganga Ram Hospital, New Delhi; Arvind Bagga, All India Institute of Medical Sciences, New Delhi; Chinmay Behera, Kalinga Institute of Medical Sciences, Bhubaneswar; Gurdeep Singh Dhooria, Dayanand Medical College & Hospital, Ludhiana; Sanjeev Gulati, Fortis Escorts Heart Institute, New Delhi; NK Hase, King Edward Memorial Hospital, Mumbai; Madhuri Kanitkar, Armed Forces Medical College, Pune; Sriram Krishnamurthy, Jawaharlal Institute of Post-graduate Medical Education & Research, Puducherry; Manish Kumar, Chacha Nehru Bal Chikitsalaya, Delhi; Amarjeet Mehta, Sawai Man Singh Hospital, Jaipur; Mahantesh Patil, KLES Dr. Prabhakar Kore Hospital & Medical Research Center, Karnataka; Saroj. K. Patnaik, Army Hospital Research and Referral, New Delhi; Ravindra Prabhu, Kasturba Medical College, Manipal; Narayan Prasad, Sanjay Gandhi Post-Graduate Institute of Medical Sciences, Lucknow; Padmaraj Rajendran, Institute of Child Health, Chennai; Abhijeet Saha, Lady Hardinge Medical College, New Delhi; VK Sairam, Kanchi Kamacoti Child Trust Hospital, Chennai; T. Saravanan, PSG Coimbatore; Sidharth Kumar Sethi, Medanta The Medicity, Gurugram; Fagun Shah, Child's Kidney Care Center, Surat; Rajiv Sinha, Institute of Child Health, Kolkata; Deepti Suri, Post-Graduate Institute of Medical Education and Research, Chandigarh; Nilam Thaker, Ahmedabad; AS Vasudev, Indraprastha Apollo Hospital, New Delhi; Anil Vasudevan, St. John's Medical College and Hospital, Bangalore; M Vijayakumar (late), Mehta Children Hospital, Chennai; Susan Uthup, Trivandrum Medical College, Thiruvananthapuram.

### Conflict of Interest Statement

The authors declare that the research was conducted in the absence of any commercial or financial relationships that could be construed as a potential conflict of interest.

## References

[1] Fakhouri F Zuber J Fremeaux-Bacchi V Loirat C. Haemolytic uraemic syndrome. Lancet. (2017) 390:681–96. 10.1016/S0140-6736(17)30062-4 28242109

[2] Noris M Remuzzi G Atypical hemolytic-uremic syndrome. N Engl J Med. (2009) 361:1676–87. 10.1056/NEJMra0902814 19846853

[3] Thergaonkar RW Narang A Gurjar BS Tiwari P Puraswani M Saini H. Targeted exome sequencing in anti-factor H antibody negative HUS reveals multiple variations. Clin Exp Nephrol. (2018) 22:653–60. 10.1007/s10157-017-1478-6 28939980

[4] Hofer J Janecke AR Zimmerhackl LB Riedl M Rosales A Giner T. Complement factor H-related protein 1 deficiency and factor H antibodies in pediatric patients with atypical hemolytic uremic syndrome. Clin J Am Soc Nephrol. (2013) 8:407–15. 10.2215/CJN.01260212 23243267 PMC3586960

[5] Jozsi M Licht C Strobel S Zipfel SL Richter H Heinen S. Factor H autoantibodies in atypical hemolytic uremic syndrome correlate with CFHR1/CFHR3 deficiency. Blood. (2008) 111:1512–4. 10.1182/blood-2007-09-109876 18006700

[6] Dragon-Durey MA Sethi SK Bagga A Blanc C Blouin J Ranchin B. Clinical features of anti-factor H autoantibody-associated hemolytic uremic syndrome. J Am Soc Nephrol. (2010) 21:2180–7. 10.1681/ASN.2010030315 21051740 PMC3014031

[7] Sinha A Gulati A Saini S Blanc C Gupta A Gurjar BS. Prompt plasma exchanges and immunosuppressive treatment improves the outcomes of anti-factor H autoantibody-associated hemolytic uremic syndrome in children. Kidney Int. (2014) 85:1151–60. 10.1038/ki.2013.373 24088957

[8] Schaefer F Ardissino G Ariceta G Fakhouri F Scully M Isbel N. Clinical and genetic predictors of atypical hemolytic uremic syndrome phenotype and outcome. Kidney Int. (2018) 94:408–18. 10.1016/j.kint.2018.02.029 29907460

[9] Durey MA Sinha A Togarsimalemath SK Bagga A. Anti-complement-factor H-associated glomerulopathies. Nat Rev Nephrol. (2016) 12:563–78. 10.1038/nrneph.2016.99 27452363

[10] Khandelwal P Gupta A Sinha A Saini S Hari P Dragon Durey MA. Effect of plasma exchange and immunosuppressive medications on antibody titers and outcome in anti-complement factor H antibody-associated hemolytic uremic syndrome. Pediatr Nephrol. (2015) 30:451–7. 10.1007/s00467-014-2948-7 25217328

[11] Jozsi M Strobel S Dahse HM Liu WS Hoyer PF Oppermann M. Anti factor H autoantibodies block C-terminal recognition function of factor H in hemolytic uremic syndrome. Blood. (2007) 110:1516–8. 10.1182/blood-2007-02-071472 17495132

[12] Strobel S Hoyer PF Mache CJ Sulyok E Liu WS Richter H. Functional analyses indicate a pathogenic role of factor H autoantibodies in atypical haemolytic uraemic syndrome. Nephrol Dial Transplant. (2010) 25:136–44. 10.1093/ndt/gfp388 19666655

[13] Blanc C Roumenina LT Ashraf Y Hyvarinen S Sethi SK Ranchin B. Overall neutralization of complement factor H by autoantibodies in the acute phase of the autoimmune form of atypical hemolytic uremic syndrome. J Immunol. (2012) 189:3528–37. 10.4049/jimmunol.1200679 22922817

[14] Nozal P Bernabeu-Herrero ME Uzonyi B Szilagyi A Hyvarinen S Prohaszka Z. Heterogeneity but individual constancy of epitopes, isotypes and avidity of factor H autoantibodies in atypical hemolytic uremic syndrome. Mol Immunol. (2016) 70:47–55. 10.1016/j.molimm.2015.12.005 26703217

[15] Loirat C Fakhouri F Ariceta G Besbas N Bitzan M Bjerre A. An international consensus approach to the management of atypical hemolytic uremic syndrome in children. Pediatr Nephrol. (2016) 31:15–39. 10.1007/s00467-015-3076-8 25859752

[16] Gurjar BS Manikanta Sriharsha T Bhasym A Prabhu S Puraswani M Khandelwal P Characterization of genetic predisposition and autoantibody profile in atypical haemolytic-uraemic syndrome. Immunology. 154:663–72. 10.1111/imm.12916 PMC6050217 29485195

[17] Schwartz GJ Munoz A Schneider MF Mak RH Kaskel F Warady BA. New equations to estimate GFR in children with CKD. J Am Soc Nephrol. (2009) 20:629–37. 10.1681/ASN.2008030287 19158356 PMC2653687

[18] Flynn JT Kaelber DC Baker-Smith CM Blowey D Carroll AE Daniels SR. Clinical practice guideline for screening and management of high blood pressure in children and adolescents. Pediatrics. (2017) 140:e20171904 . 10.1542/peds.2017-1904 28827377

[19] Kidney Disease: Improving Global Outcomes (KDIGO) CKD Work Group KDIGO 2012 clinical practice guideline for the evaluation and management of chronic kidney disease. Kidney Int Suppl. (2013) 3:1–150. 10.1038/kisup.2012.64

[20] Khoury PR Mitsnefes M Daniels SR Kimball TR. Age-specific reference intervals for indexed left ventricular mass in children. J Am Soc Echocardiogr. (2009) 22:709–14. 10.1016/j.echo.2009.03.003 19423289

[21] Expert Panel on Integrated Guidelines for Cardiovascular Health and Risk Reduction in Children and Adolescents; National Heart Lung and Blood Institute Expert panel on integrated guidelines for cardiovascular health and risk reduction in children and adolescents: summary report. Pediatrics. (2011) 128(Suppl. 5):S213–56. 10.1542/peds.2009-2107C 22084329 PMC4536582

[22] Flynn JT Daniels SR Hayman LL Maahs DM McCrindle BW Mitsnefes M. Update: ambulatory blood pressure monitoring in children and adolescents: a scientific statement from the American Heart Association. Hypertension. (2014) 63:1116–35. 10.1161/HYP.0000000000000007 24591341 PMC4146525

[23] Delanaye P Mariat C Cavalier E Maillard N Krzesinski JM White CA. Trimethoprim, creatinine and creatinine-based equations. Nephron Clin Pract. (2011) 119:c187–93. Discussion c93–4. 10.1159/000328911 21832843

[24] Hellerstein S Berenbom M Erwin P Wilson N DiMaggio S. Measurement of renal functional reserve in children. Pediatr Nephrol. (2004) 19:1132–6. 10.1007/s00467-004-1550-9 15258846

[25] Brocklebank V Johnson S Sheerin TP Marks SD Gilbert RD Tyerman K. Factor H autoantibody is associated with atypical hemolytic uremic syndrome in children in the United Kingdom and Ireland. Kidney Int. (2017) 92:1261–71. 10.1016/j.kint.2017.04.028 28750931 PMC5652378

[26] Togarsimalemath SK Si-Mohammed A Puraswani M Gupta A Vabret A Liguori S. Gastrointestinal pathogens in anti-FH antibody positive and negative Hemolytic Uremic Syndrome. Pediatr Res. (2018) 84:118–24. 10.1038/s41390-018-0009-9 29795200

[27] Jozsi M Oppermann M Lambris JD Zipfel PF. The C-terminus of complement factor H is essential for host cell protection. Mol Immunol. (2007) 44:2697–706. 10.1016/j.molimm.2006.12.001 17208302 PMC2700862

[28] Sanchez-Corral P Gonzalez-Rubio C Rodriguez de Cordoba S Lopez-Trascasa M. Functional analysis in serum from atypical Hemolytic Uremic Syndrome patients reveals impaired protection of host cells associated with mutations in factor H. Mol Immunol. (2004) 41:81–4. 10.1016/j.molimm.2004.01.003 15140578

[29] Song D Liu XR Chen Z Xiao HJ Ding J Sun SZ. The clinical and laboratory features of Chinese Han anti-factor H autoantibody-associated hemolytic uremic syndrome. Pediatr Nephrol. (2017) 32:811–22. 10.1007/s00467-016-3562-7 28035470

[30] Khandelwal P Sinha A Hari P Bansal VK Dinda AK Bagga A. Outcomes of renal transplant in patients with anti-complement factor H antibody-associated hemolytic uremic syndrome. Pediatr Transplant. (2014) 18:E134–9. 10.1111/petr.12273 24814615

[31] Nozal P Garrido S Alba-Dominguez M Espinosa L Pena A Cordoba SR. An ELISA assay with two monoclonal antibodies allows the estimation of free factor H and identifies patients with acquired deficiency of this complement regulator. Mol Immunol. (2014) 58:194–200. 10.1016/j.molimm.2013.11.021 24378252

[32] Strobel S Abarrategui-Garrido C Fariza-Requejo E Seeberger H Sanchez-Corral P Jozsi M. Factor H-related protein 1 neutralizes anti-factor H autoantibodies in autoimmune hemolytic uremic syndrome. Kidney Int. (2011) 80:397–404. 10.1038/ki.2011.152 21677636

[33] Bu F Meyer NC Zhang Y Borsa NG Thomas C Nester C. Soluble c5b-9 as a biomarker for complement activation in atypical hemolytic uremic syndrome. Am J Kidney Dis. (2015) 65:968–9. 10.1053/j.ajkd.2015.02.326 25818678

[34] Cataland SR Holers VM Geyer S Yang S Wu HM. Biomarkers of terminal complement activation confirm the diagnosis of aHUS and differentiate aHUS from TTP. Blood. (2014) 123:3733–8. 10.1182/blood-2013-12-547067 24695849

[35] Volokhina EB Westra D van der Velden TJ van de Kar NC Mollnes TE van den Heuvel LP. Complement activation patterns in atypical haemolytic uraemic syndrome during acute phase and in remission. Clin Exp Immunol. (2015) 181:306–13. 10.1111/cei.12426 25079699 PMC4516446

[36] Noris M Galbusera M Gastoldi S Macor P Banterla F Bresin E. Dynamics of complement activation in aHUS and how to monitor eculizumab therapy. Blood. (2014) 124:1715–26. 10.1182/blood-2014-02-558296 25037630 PMC4162105

[37] Bagga A Khandelwal P Mishra K Thergaonkar R Vasudevan A Sharma J. Hemolytic uremic syndrome in a developing country: consensus guidelines. Pediatr Nephrol. (2019). 10.1007/s00467-019-04233-7. [Epub ahead of print]. 30989342

[38] Bhattacharjee A Reuter S Trojnar E Kolodziejczyk R Seeberger H Hyvarinen S. The major autoantibody epitope on factor H in atypical hemolytic uremic syndrome is structurally different from its homologous site in factor H-related protein 1, supporting a novel model for induction of autoimmunity in this disease. J Biol Chem. (2015) 290:9500–10. 10.1074/jbc.M114.630871 25659429 PMC4392255

[39] Moore I Strain L Pappworth I Kavanagh D Barlow PN Herbert AP. Association of factor H autoantibodies with deletions of CFHR1, CFHR3, CFHR4, and with mutations in CFH, CFI, CD46, and C3 in patients with atypical hemolytic uremic syndrome. Blood. (2010) 115:379–87. 10.1182/blood-2009-05-221549 19861685 PMC2829859

[40] Guo WY Song D Liu XR Chen Z Xiao HJ Ding J. Immunological features and functional analysis of anti-CFH autoantibodies in patients with atypical hemolytic uremic syndrome. Pediatr Nephrol. (2019) 34:269–81. 10.1007/s00467-018-4074-4 30315407

[41] Ariceta G Besbas N Johnson S Karpman D Landau D Licht C. Guideline for the investigation and initial therapy of diarrhea-negative hemolytic uremic syndrome. Pediatr Nephrol. (2009) 24:687–96. 10.1007/s00467-008-0964-1 18800230

[42] Johnson S Stojanovic J Ariceta G Bitzan M Besbas N Frieling M. An audit analysis of a guideline for the investigation and initial therapy of diarrhea negative (atypical) hemolytic uremic syndrome. Pediatr Nephrol. (2014) 29:1967–78. 10.1007/s00467-014-2817-4 24817340

[43] Khandelwal P Thomas CC Rathi BS Hari P Tiwari AN Sinha A Membrane-filtration based plasma exchanges for atypical hemolytic uremic syndrome: Audit of efficacy and safety. J Clin Apher. (in press). 10.1002/jca.21711 31173399

[44] Goodship TH Cook HT Fakhouri F Fervenza FC Fremeaux-Bacchi V Kavanagh D. Atypical hemolytic uremic syndrome and C3 glomerulopathy: conclusions from a “Kidney Disease: improving Global Outcomes” (KDIGO) Controversies Conference. Kidney Int. (2017) 91:539–51. 10.1016/j.kint.2016.10.005 27989322

[45] Spinale JM Ruebner RL Copelovitch L Kaplan BS. Long-term outcomes of Shiga toxin hemolytic uremic syndrome. Pediatr Nephrol. (2013) 28:2097–105. 10.1007/s00467-012-2383-6 23288350

[46] Krmar RT Ferraris JR Ramirez JA Ruiz S Salomon A Galvez HM. Ambulatory blood pressure monitoring after recovery from hemolytic uremic syndrome. Pediatr Nephrol. (2001) 16:812–6. 10.1007/s004670100679 11605788

[47] De Petris L Gianviti A Giordano U Calzolari A Tozzi AE Rizzoni G. Blood pressure in the long-term follow-up of children with hemolytic uremic syndrome. Pediatr Nephrol. (2004) 19:1241–4. 10.1007/s00467-004-1582-1 15322891

[48] Kreuzer M Sollmann L Ruben S Leifheit-Nestler M Fischer DC Pape L. Endothelial dysfunction during long-term follow-up in children with STEC hemolytic-uremic syndrome. Pediatr Nephrol. (2017) 32:1005–11. 10.1007/s00467-016-3574-3 28180952

[49] Lurbe E Torro MI Alvarez J. Ambulatory blood pressure monitoring in children and adolescents: coming of age? Curr Hypertens Rep. (2013) 15:143–9. 10.1007/s11906-013-0350-7 23591725

[50] Lurbe E Redon J Kesani A Pascual JM Tacons J Alvarez V. Increase in nocturnal blood pressure and progression to microalbuminuria in type 1 diabetes. N Engl J Med. (2002) 347:797–805. 10.1056/NEJMoa013410 12226150

[51] Perelstein EM Grunfield BG Simsolo RB Gimenez MI Gianantonio CA. Renal functional reserve compared in haemolytic uraemic syndrome and single kidney. Arch Dis Child. (1990) 65:728–31. 2386378 10.1136/adc.65.7.728 PMC1792424

[52] Tufro A Arrizurieta EE Repetto H. Renal functional reserve in children with a previous episode of haemolytic-uraemic syndrome. Pediatr Nephrol. (1991) 5:184–8. 2031831 10.1007/BF01095948

[53] Dieguez S Ayuso S Brindo M Osinde E Canepa C. Renal functional reserve evolution in children with a previous episode of hemolytic uremic syndrome. Nephron Clin Pract. (2004) 97:c118–22. 10.1159/000078640 15292689

[54] Bruno GO Dieguez SM Voyer LE. [Renal functional reserve in children with a history of hemolytic uremic syndrome through technetium-99m diethylene-triamine-penta-acetic acid clearance]. Arch Argent Pediatr. (2012) 110:60–3. 10.1590/S0325-00752012000100012 22307424

[55] Gabbai FB. The role of renal response to amino acid infusion and oral protein load in normal kidneys and kidney with acute and chronic disease. Curr Opin Nephrol Hypertens. (2018) 27:23–9. 10.1097/MNH.0000000000000380 29068795

[56] Gaipov A Solak Y Zhampeissov N Dzholdasbekova A Popova N Molnar MZ. Renal functional reserve and renal hemodynamics in hypertensive patients. Ren Fail. (2016) 38:1391–7. 10.1080/0886022X.2016.1214052 27470640

[57] Livi R Guiducci S Perfetto F Ciuti G Grifoni E Conforti L. Lack of activation of renal functional reserve predicts the risk of significant renal involvement in systemic sclerosis. Ann Rheum Dis. (2011) 70:1963–7. 10.1136/ard.2011.152892 21784725

