# Supplementary material for: Clinical and Immunological Profile of Anti-factor H Antibody Associated Atypical Hemolytic Uremic Syndrome: A Nationwide Database
Source: Front Immunol. 2019 Jun 7;10:1282. doi: 10.3389/fimmu.2019.01282 (PMC6567923; doi:10.3389/fimmu.2019.01282)
Supplement: Supplementary file 1 [file Table_1.DOCX]

**Supplementary tables**

**Supplementary Table 1** Epitope specificity of anti-factor H antibodies during onset, remission and relapse

| Patient | Disease state | SCR 1-4 | SCR 5-8 | SCR 9-12 | SCR 13-16 | SCR 17-20 | SRBC hemolysis (%) |
| --- | --- | --- | --- | --- | --- | --- | --- |
| 1 | Onset | - | + | ++ | - | +++ | 45 |
|  | Remission | - | + | + | - | +++ | <20 |
| 2^ | Onset | - | - | ++ | ++ | +++ | 82 |
|  | Remission | - | - | ++ | ++ | +++ | <20 |
| 3 | Onset | - | + | - | ++ | +++ | 69.4 |
|  | Remission | - | + | - | ++ | +++ | <20 |
| 4 | Onset | + | + | ++ | ++ | +++ | 56.5 |
|  | Remission | + | + | ++ | ++ | +++ | <20 |
| 5 | Onset | - | - | ++ | ++ | +++ | 40 |
|  | Relapse | - | - | ++ | ++ | +++ | 53 |
| 6 | Onset | - | - | ++ | ++ | +++ | 75.7 |
|  | Relapse | - | + | ++ | ++ | +++ | 81.3 |
| 7 | Onset | + | + | ++ | ++ | +++ | 67 |
|  | Remission | + | + | ++ | ++ | +++ | <20 |
|  | Relapse | + | + | ++ | ++ | +++ | 40 |
| 8 | Onset | + | - | + | + | +++ | 85 |
|  | Remission | + | - | + | + | +++ | <20 |

*SCR* short consensus repeats; *SRBC* sheep red blood cell

Percentage of binding to respective fragments was graded as 10-30% (+), 30-70% (++) and >70% (+++)

^All patients showed homozygous deletion of *CFHR1* except patient #2 who had normal *CFHR1*

**Supplementary Table 2** Outcome of patients managed with combined plasma-exchanges and immunosuppression, N=196

| **Outcome** | **At 3 months** | **At last follow up** |
| --- | --- | --- |
| CKD stage 1 (estimated GFR ≥90 mL/min/1.73 m^2^)  With normal urinalysis  With hypertension stage 2, hematuria or proteinuria ≥2+  CKD stages 2-3 (estimated GFR 30-89 mL/min/1.73 m^2^)  Adverse outcome (CKD stage 4-5; patient death) | 27 (13.8%)  108 (55.1%)  37 (18.9%)  24 (12.2%) | 82 (41.8%)  64 (32.7%)  23 (11.7%)  27 (13.8%) |

Adverse outcome includes 7 and 12 patient deaths at 3-months and at last follow up, respectively

CKD chronic kidney disease, GFR glomerular filtration rate

**Supplementary Table 3** Determinants of adverse outcome, defined by chronic kidney disease stage 4-5 (estimated glomerular filtration rate <30 ml/min/1.73 m^2^) or patient death at last follow up

| Parameter, N=356 | Univariate analysis | | Multivariable analysis | |
| --- | --- | --- | --- | --- |
|  | Hazards ratio (95%CI) | P | Hazards ratio (95%CI) | P |
| Age, years | 1.02 (0.96, 1.09) | 0.73 | - | - |
| Duration of oliguria > 7 days | 2.47 (1.57, 3.88) | <0.001 | 1.74 (0.80, 3.78) | 0.16 |
| Neurological manifestations | 1.47 (0.96, 2.24) | 0.07 | 1.89 (0.91, 3.98) | 0.092 |
| C3 <70 mg/dl | 2.30 (1.43, 3.70) | 0.001 | 1.66 (0.76, 3.62) | 0.20 |
| Anti-factor H antibody ≥8000 AU/ml | 1.68 (1.08, 2.62) | 0.021 | 2.23 (1.11, 4.48) | 0.024 |
| Requirement of dialysis | 4.02 (1.75, 9.21) | 0.001 | 1.68 (0.37, 7.60) | 0.50 |
| Plasma exchange (PEX) <14 days | 2.70 (1.54, 4.73) | 0.001 | 2.60 (1.19, 5.67) | 0.017 |
| Time to PEX ≥14 days | 1.64 (0.97, 2.80) | 0.064 | 2.09 (0.93, 4.69) | 0.071 |
| Immunosuppression ± plasma infusion | 0.60 (0.24, 1.49) | 0.27 | - | - |
| PEX, steroid & immunosuppression | 0.27 (0.17, 0.43) | <0.001 | 0.37 (0.16, 0.89) | 0.026 |
| Maintenance immunosuppression* | 0.07 (0.02, 0.28) | <0.001 | 0.02 (0.001, 0.413) | 0.011 |

*Assessed in patients with favorable renal outcome at 3 months
