# Supplementary material for: Clinical and Immunological Profile of Anti-factor H Antibody Associated Atypical Hemolytic Uremic Syndrome: A Nationwide Database
Source: Front Immunol. 2019 Jun 7;10:1282. doi: 10.3389/fimmu.2019.01282 (PMC6567923; doi:10.3389/fimmu.2019.01282)
Supplement: Supplementary file 2 [file Table_2.DOCX]

**Legends to Supplementary Figures:**

**Fig. 1.** Map of India, showing origin of patients with atypical hemolytic uremic syndrome who were positive for anti-factor H antibodies.

**Fig. 2.** Number of patients with (solid bars) and without (dotted lines) anti-factor H antibody associated HUS, according to age at presentation

**Fig. 3.** Number of patients with (solid bars) and without (dotted lines) anti-factor H antibody associated HUS, with onset of illness in relation to month of the year

**Fig. 4.** Scatter-plot showing significant correlation between anti-factor H (FH) antibody titers at onset and disease severity as indicated by serum C3 **(A)**, lowest platelet count **(B)**, lowest hemoglobin level **(C)** and peak lactate dehydrogenase (LDH; **D**). Anti-FH antibody titers are plotted on a logarithmic scale.

**Fig. 5.** Functional renal reserve test in 41 patients. Bars represent baseline estimated glomerular filtration rate (eGFR, blue) and increase in urinary creatinine clearance over and above the basal creatinine clearance (renal reserve, orange) in each patient following an oral protein load
