# Supplementary figures and images for: Clinical and Immunological Profile of Anti-factor H Antibody Associated Atypical Hemolytic Uremic Syndrome: A Nationwide Database
Source: Front Immunol. 2019 Jun 7;10:1282. doi: 10.3389/fimmu.2019.01282 (PMC6567923; doi:10.3389/fimmu.2019.01282)

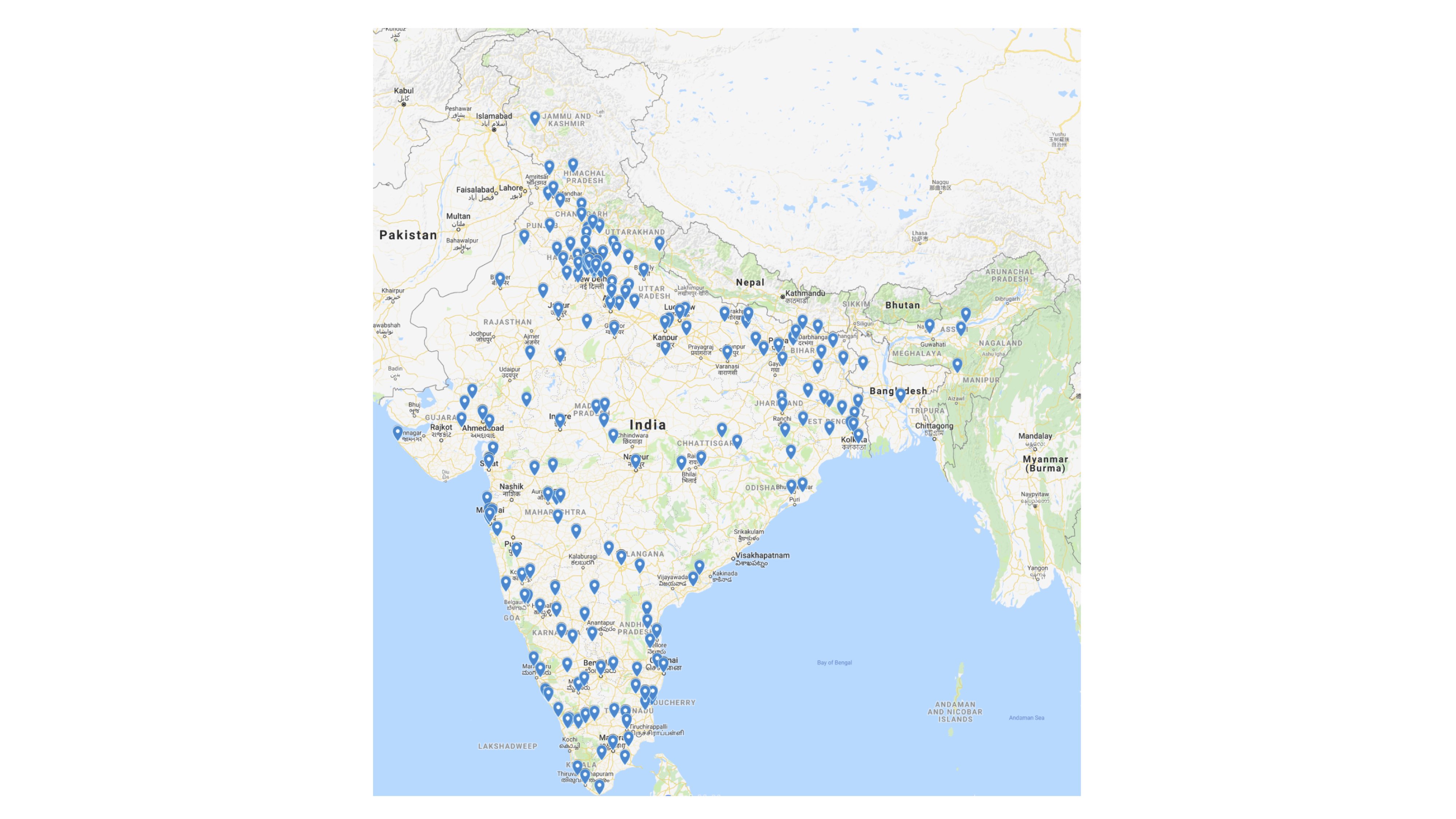

Supplement: Supplementary file 3 [file Image_1.JPEG]

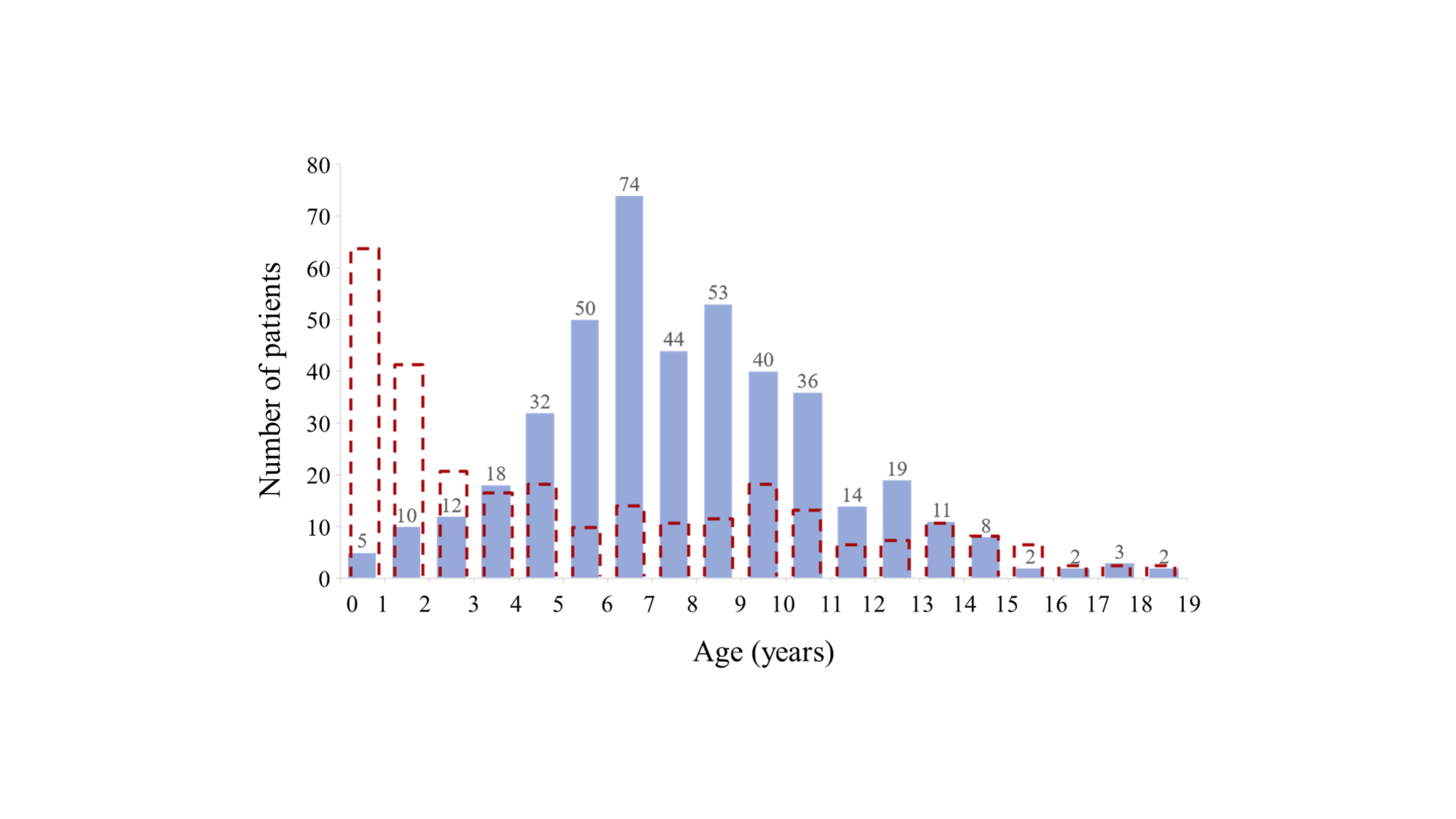

Supplement: Supplementary file 4 [file Image_2.JPEG]

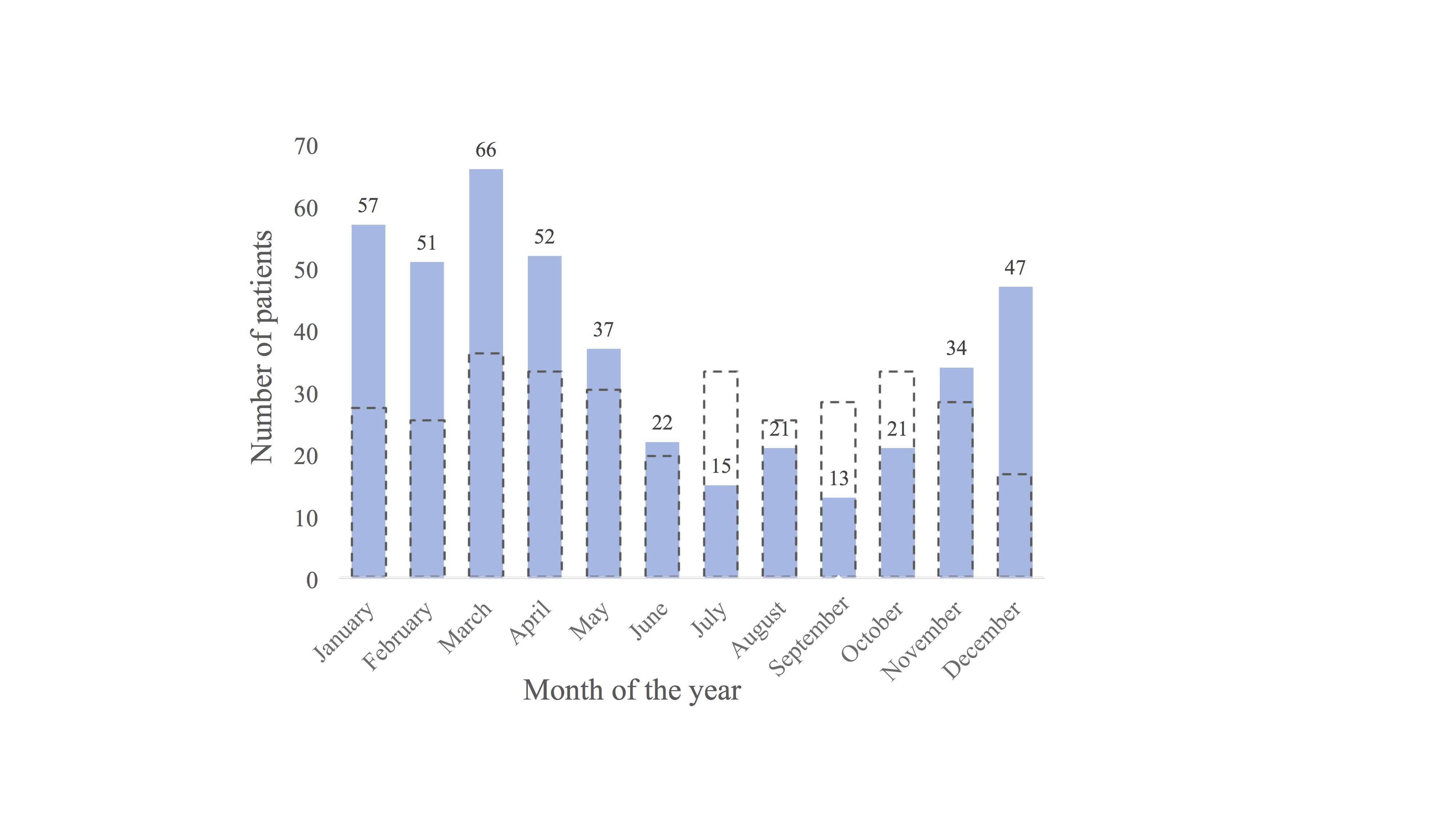

Supplement: Supplementary file 5 [file Image_3.JPEG]

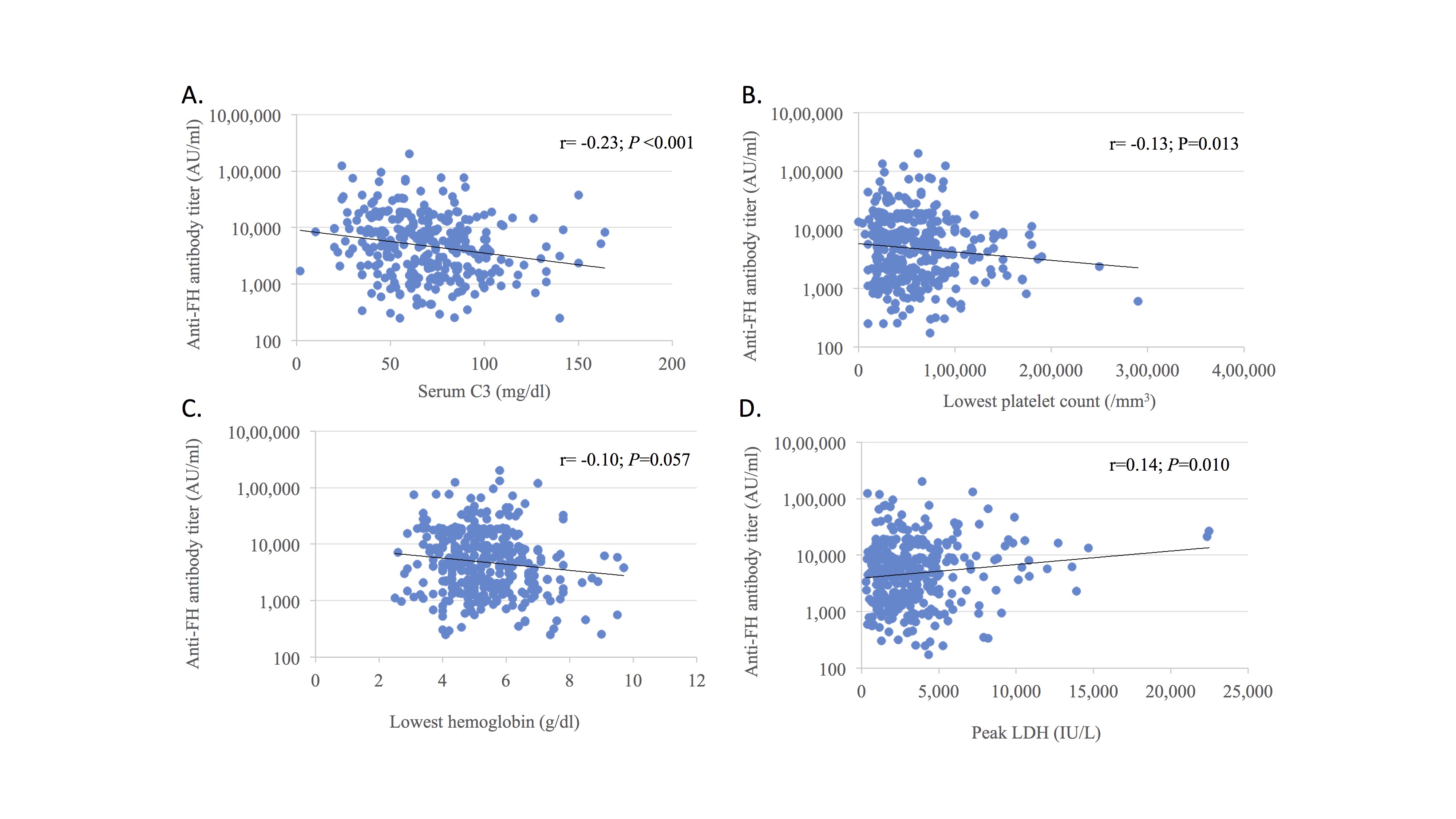

Supplement: Supplementary file 6 [file Image_4.JPEG]

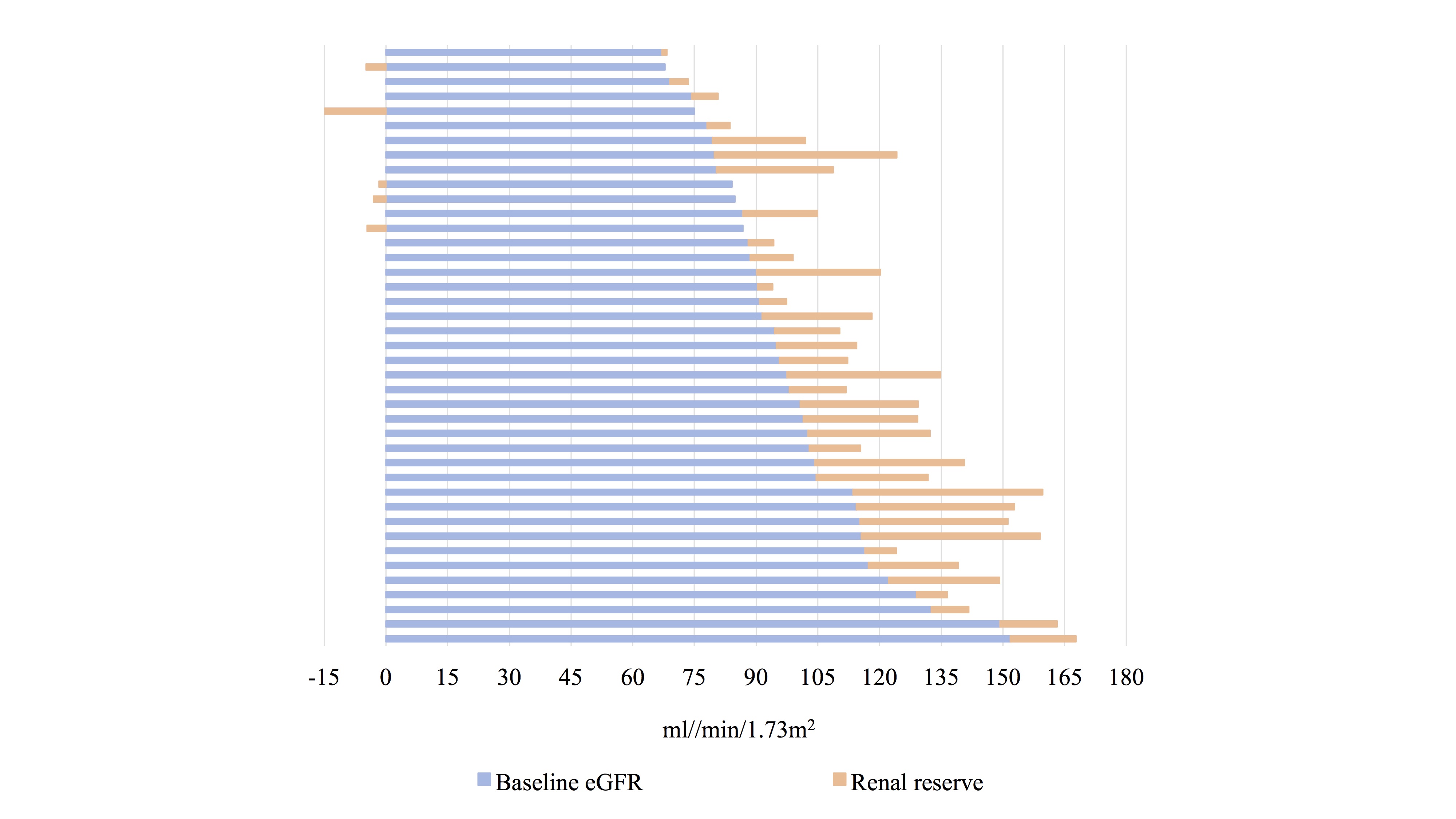

Supplement: Supplementary file 7 [file Image_5.JPEG]
